# Impact of maternal obesity on the incidence of pregnancy complications in France and Canada

**DOI:** 10.1038/s41598-017-11432-5

**Published:** 2017-09-07

**Authors:** Florent Fuchs, Marie-Victoire Senat, Evelyne Rey, Jacques Balayla, Nils Chaillet, Jean Bouyer, François Audibert

**Affiliations:** 1 Department of Obstetrics and Gynecology, CHU Sainte Justine, Montréal, Québec, Canada; 2 Inserm, CESP Centre for research in Epidemiology and Population Health, U1018, Reproduction and child development, Villejuif, France; 3 Department of Obstetrics and Gynecology. Hôpital Bicêtre, Assistance Publique Hôpitaux de Paris (APHP), Le Kremlin-Bicêtre, France; 4 Division of Obstetric Medicine, Department of Obstetrics and Gynecology, CHU Sainte Justine, Montréal, Québec, Canada; 5 Clinical Research Center Étienne-Le Bel, CHU Sherbrooke, Sherbrooke Québec, Canada

## Abstract

The aim of our study was to compare the impact of maternal obesity on the incidence of medical complications of pregnancy in France and Canada. We performed a prospective comparative cohort study using French data, retrieved from a prospective cohort of singleton deliveries, and Canadian data retrieved from QUARISMA, a cluster-randomized controlled trial conducted in Quebec, both between 2009 and 2011. Outcomes studied included, hypertensive disorders of pregnancy (HDP), venous thromboembolism, stillbirth, caesarean delivery and macrosomia. The impact of obesity across both cohorts was studied using univariate and multivariate logistic regression analyses, adjusting for relevant confounders. The French and Canadian databases included 26,973 and 22,046 deliveries respectively, with obesity rates of 9.1% and 16% respectively (p < 0.001). In both cohorts, obesity was significantly associated with an increased rate of HDP, cesarean delivery, and macrosomia. However, in both cohorts the relationship between increasing body mass index and the incidence of medical complication of pregnancy was the same, regardless the outcome studied. In conclusion, obesity is a risk factor for adverse maternal and fetal outcomes in both cohorts. Similar trends of increased risk were noted in both cohorts even though obesity is more prevalent in Canada.

## Introduction

The prevalence of obesity has dramatically increased over the past 30 years, especially in North America. This condition is now described as an “epidemic”, and has consistently been associated to adverse health outcomes such as cardiovascular disorders, metabolic disorders, the occurrence of certain cancers, as well as psychosocial problems^1^. The impact of obesity on life expectancy is considerable: in nonsmoking Americans, obesity at 40 years of age is associated with a life expectancy reduction of 7.1 years for women and 5.8 years for men^2^. In Canada, the rate of obesity among adult women of reproductive age has increased from 16% in 1997, to 23.9% in 2009 (+50%) whereas the increase has been 82% in the same period in France, from 8.3% to 15.1%^3–5^. A similar trend has been observed in pregnant women: in 2009, 16% of women starting a pregnancy were obese in Canada, in comparison to 9.9% in France^6, 7^. This expansion is both linked to a general trend of increasing obesity but also to an increase in the age of childbearing in recent years; indeed, obesity also increases with advancing maternal age^4, 7^. Consequently, obesity appears to be more longstanding and more prevalent in Canada than in France.

Definitions of obesity have varied widely over time. The most frequent definition utilized is based on the Body Mass Index (BMI), a simple measure of weight-for-height defined as the weight in kilograms divided by the square of the height in meters (kg/m^2^). The ranges are independent of age, parity, smoking history, race and ethnic background^8–10^.

Given the growing prevalence of increased BMI and the difference in obesity rates between Canada and France, we aimed to study the relationship between obesity and the incidence of medical complications of pregnancy in those two countries. We therefore compared pregnancy outcomes according to BMI classes between the two countries, through a secondary analysis of data collected in two well-defined contemporaneous cohorts.

## Materials and Methods

The French data was retrieved from four tertiary care centers in the area of Paris, France, each performing more than 2500 deliveries a year. All singleton deliveries from January 1^st^, 2009 to December 31^st^, 2011, with a gestational age of 24 weeks or beyond, and a systematically recorded early pregnancy BMI value, were anonymously extracted from a computerized database (DIAMM®, micro6©) in each center by an independent data abstractor. All maternal and neonatal data were collected and prospectively registered in this database by the medical team in charge of the patients. DIAMM is a database approved by CNIL, the French Data Protection Authority, under the notification number 1181076. After completion of the data in each hospital, data were merged to create our working French cohort, which included 26,973 deliveries.

The Canadian data was obtained from the QUARISMA randomized controlled trial^11^. QUARISMA was a cluster intervention trial designed to assess the effectiveness of a complex intervention with background information and audits targeting a low-risk population in terms of safe and sustainable reduction in the rate of caesarean sections. It took place in 32 hospitals in the province of Quebec, Canada, from 2009 to 2011 and enabled to collect information on more than 185,000 pregnancies. Since the QUARISMA intervention has demonstrated its effectiveness in the reduction of cesarean delivery, we restricted our analysis to women in the observational arm of the trial. These women carried a singleton pregnancy, with a gestational age of 24 weeks or beyond, and a recorded pre-pregnancy BMI value, who delivered in the four tertiary care center of the province of Quebec. Comparison of patient characteristics between the group with available BMI information and that without revealed no major differences. Overall, the Canadian study population included 22,046 deliveries. The QUARISMA study was approved by the institutional review boards of the 32 hospitals included in the trial.

The current study was approved by the French institutional review board of the «Comité d’éthique de la recherche en obstétrique et gynécologie» (IRB Study Number CEROG OBS 2013-06-04). Requirement for individual informed consent was waived by the ethics committee.

For both cohorts, demographic characteristics, obstetrical history, pregnancy and neonatal data were included in the database and compared. The BMI value was obtained in each cohort by measuring height and weight at early pregnancy prenatal visits. The medical complications of pregnancy studied included: hypertensive disorders of pregnancy, venous thromboembolic disease, stillbirth, caesarean delivery, and macrosomia. Hypertensive disorders of pregnancy is an all-encompassing term, which includes gestational hypertension, preeclampsia, severe preeclampsia, and eclampsia. Gestational hypertension was defined by a systolic pressure of 140 mm Hg or higher or a diastolic pressure of 90 mm Hg or higher on two separate occasions after 20 weeks of gestation in the absence of proteinuria. Preeclampsia was defined as gestational hypertension with either proteinuria 300 mg or more in a 24-hour sample or, if a 24-hour sample was not available, 2+ or higher on dipstick testing, or a urinary protein-to-creatinine ratio of 0.03 g/mmol or more^12, 13^. Severe preeclampsia was defined as preeclampsia associated with any adverse criteria: systolic pressure of 160 mm Hg or higher or a diastolic pressure of 110 mm Hg, or renal impairment (oliguria < 500 mL/24 hours, or creatinine > 135 micromol/L, or proteinuria > 5 g/24 hours), or pulmonary edema, or thrombocytopenia < 100 G/L, or eclampsia. Macrosomia was defined as a neonatal birthweight of 4000 g or above, and severe macrosomia when birthweight exceeded 4500 g. Some other characteristics such as “chronic disease” were also reported in both cohorts to be used as covariates.

Following, the data was stratified by cohort and early pregnancy BMI was categorized according to the World Health Organisation’s (WHO) definition: underweight (less than 18.5 kg/m²), normal weight (18.5–24.9 kg/m²), overweight (25–29.9 kg/m²), class I obesity (30-34.9 kg/m²), class II obesity (35–39.9 kg/m²), and class III obesity (equal or more than 40 kg/m²). The relationship between early-pregnancy BMI category and the incidence of each outcome was calculated using Student t-tests for continuous data, and Fisher’s exact or χ2 tests for categorical data. Univariate analysis computed crude odds ratio (OR), with the reference being the normal weight BMI class (18.5–24.9 kg/m²). Before calculating OR for both cohorts, an interaction test (χ2 test of homogeneity) between the cohort and BMI for a particular outcome variable was assessed. When the interaction test was significant, ORs stratified by cohort were calculated, whereas a single Mantel Haenzel OR was computed in the absence of interaction. Multiple logistic regression analyses were used to assess the specific influence of BMI categories (adjusted odds ratio: aOR) following adjustment for pertinent covariates^14^. Various adjustments were made on known risks factors retrieved from the literature, according to the outcome studied. As in univariate analyses, the reference for multivariate analyses was the normal weight BMI class. In a separate analysis, we decided to describe the relationship between BMI and the different maternal fetal outcomes studied, using fractional polynomial (PF) modeling, since BMI can be defined as a continuous variable^15^. This modeling produces a graph of the curve that best fitted the relation between BMI and the different outcomes studied. Therefore, we computed a graph for each cohort, describing the relationship between BMI and the studied outcome. In addition to using the PF modeling of BMI, we performed univariate analyses and then multiple logistic regression analyses for both countries to evaluate the interactions between the studied cohort and the different BMI categories. Then, using this PF modeling we computed aOR for fixed BMI values that corresponded to the median value of BMI inside each category considered. As explained before, when the interaction test (using PF modeling of BMI) was not statistically significant, a single aOR was reported. Finally, due to the common nature of some of the outcome studies, such as cesarean delivery, odds ratios were not appropriate enough to express the impact of obesity^16^. Risk ratios (RR) and adjusted risk ratios (aRR) were then estimated in every model (crude and adjusted). To clarify our results, only RR and aRR are presented in the tables. By convention, results were considered statistically significant when p < 0.05. Statistical analyses were performed with STATA software, v.13 (Stata Corporation, College Station, TX).

## Results

The French group included 26,973 singleton deliveries, whereas the Canadian group consisted of 22,046 singleton deliveries. The prevalence of obesity was 9.1% in France and 16% in Canada (p < 0.001). Baseline characteristics are presented in Table 1. BMI category distribution was as follows (France vs. Canada): underweight: 8.0% vs. 2.5%; normal weight: 64.3% vs. 58%; overweight: 18.6% vs. 23.5%; class I obesity: 6.2% vs. 9.8%; class II obesity: 2.0% vs. 3.9%; and class III obesity: 0.9% vs. 2.3% (p < 0.001). The mean BMI was significantly lower in France than in Canada (23.2 vs. 25.1, p < 0.001). Irrespective of BMI categories, French women were older than Canadian women (p < 0.001), and more frequently nulliparous (p < 0.001). However, tobacco-smoking rates were lower in French rather than Canadian women (p < 0.001). In France, smoking was not correlated with BMI class except for underweight women, whereas underweight and Class I obese women in Canada smoked significantly more tobacco than women with a normal BMI. In both cohorts, obese women had significantly more previous hypertensive disorders in pregnancy, chronic hypertension and type 2 diabetes than women with normal BMI’s (p < 0.01). French women had a trend towards more chronic hypertension and significantly more type 1 diabetes than did Canadian women. Frequency of type 1 diabetes also increased significantly with increasing BMI categories but only in Canada (p < 0,05). Other chronic diseases such as systemic lupus erythematosus, cardiac disease, inherited thrombophilia, previous thromboembolic event, chronic kidney disease, and Crohn’s disease were more frequent in Canadian women (6.0 vs. 4.2%; p < 0.05), but no trend was observed with increasing BMI categories in neither countries.

**Table 1:** Baseline and delivery characteristics.

	FRENCH COHORT (N = 26 973)							CANADIAN COHORT (N = 22 046)						
	Total	Underweight N = 2 180 (8.0%)	Normal Weight N = 17 330 (64.3%)	Overweight N = 5 007 (18.6%)	Obesity N = 2456 (9.1%)			Total	Underw-eight N = 547 (2.5%)	Normal Weight N = 12 792 (58.0%)	Overweight N = 5 182 (23.5%)	Obesity N = 3525 (16%)		
					Class I N = 1 659 (6.2%)	Class II N = 545 (2.0%)	Class III N = 252 (0.9%)					Class I N = 2 156 (9.8%)	Class II N = 864 (3.9%)	Class III N = 505 (2.3%)
Maternal Age (y)	31.9 ± 5.5	31.4 ± 5.7^†^	31.9 ± 5.5	32.1 ± 5.6^†^	32.3 ± 5.6^†^	32.3 ± 5.6	32.4 ± 5.7	29.6 ± 4.8^§^	28.0 ± 4.7^†§^	29.5 ± 4.7^§^	29.9 ± 5.0^†§^	29.8 ± 4.9^†§^	29.9 ± 4.7^†§^	30.4 ± 4.8^†§^
Nulliparity	14489 (51.2)	1182 (54.3)	9238 (53.4)	2205 (44.1)^†^	667 (40.3)^†^	222 (40.9)^†^	102 (40.5)^†^	10105 (45.8)^§^	260 (47.5)^§^	6320 (49.4)^§^	2235 (43.1)^†^	811 (36.6)^†^	305 (33.3)^†§^	174 (34.5)^†^
Smoker	2139 (10.4)	222 (12.8)^†^	1416 (10.6)	343 (10.9)	105 (10.3)	38 (11.7)	15 (9.5)	2961(13.4)^§^	123 (22.5)^†§^	1624 (12.7)^§^	691 (13.3)^§^	323 (15.0)^†§^	126 (14.6)	74 (14.7)
Prepregnancy BMI	23.2 ± 4.7	17.6 ± 0.7^†^	21.4 ± 1.7	26.7 ± 1.4^†^	31.7 ± 1.4^†^	36.7 ± 1.4^†^	44.3 ± 5.3^†^	25.1 ± 5.6^§^	17.2 ± 0.8^†§^	21.8 ± 1.8^§^	27.1 ± 1.4^†§^	32.1 ± 1.4^†§^	37.1 ± 1.4^†§^	44.6 ± 4.7^†^
History of hypertensive disorders of pregnancy*	105 (0.5)	4 (0.2)	50 (0.4)	25 (0.7)^†^	11 (0.9)^†^	5 (1.3)^†^	4 (2.2)^†^	775 (3.5)^§^	12 (2.2)^§^	247 (1.9)^§^	232 (4.5)^†§^	143 (6.6)^†§^	86 (10)^†§^	55 (10.9)^†§^
Chronic hypertension	492 (1.7)	13 (0.6)^†^	184 (1.1)	134 (2.7)^†^	67 (4.0)^†^	36 (6.6)^†^	28 (11.1)^†^	360 (1.6)	4 (0.7)	99 (0.8)^§^	80 (1.5)^†§^	72 (3.3)^†^	52 (6.0)^†^	53 (10.5)^†^
Chronic pathology°	898 (4.2)	71 (4.3)	534 (4.1)	176 (4.8)	54 (4.4)	23 (5.7)	12 (6.2)	1316 (6.0)^§^	42 (7.7)^§^	748 (5.9)^§^	302 (5.8)^§^	128 (5.9)	64 (7.4)	32 (6.3)
Type 1 Diabetes	312 (1.1)	15 (0.7)	198 (1.1)	53 (1.1)	23 (1.4)	11 (2.0)	4 (1.6)	147 (0.7)^§^	3 (0.6)	58 (0.5)^§^	42 (0.8)^†^	31 (1.4)^†^	5 (0.6)^§^	8 (1.6)^†^
Type 2 Diabetes	108 (0.4)	4 (0.2)	25 (0.1)	29 (0.6)^†^	23 (1.4)^†^	12 (2.2)^†^	10 (4.0)^†^	96 (0.4)	2 (0.4)	16 (0.1)	28 (0.5)^†^	22 (1.0)^†^	13 (1.5)^†^	15 (3.0)^†^
Gestational age at delivery (weeks)	38.8 ± 3.1	38.8 ± 2.8	38.9 ± 3.0	38.7 ± 3.1^†^	38.3 ± 3.6^†^	38.4 ± 3.2^†^	38.2 ± 3.4^†^	38.8 ± 1.8^§^	38.5 ± 2.0^†§^	38.8 ± 1.8^§^	38.9 ± 1.8^†§^	38.7 ± 1.9^†§^	38.6 ± 2.0^†§^	38.5 ± 1.9^†§^
Birth weight (g)	3174 ± 718	3082 ± 662^†^	3181 ± 687	3226 ± 741^†^	3180 ± 828	3184 ± 804	3157 ± 842	3360 ± 539^§^	3145 ± 542^†§^	3326 ± 519^§^	3421 ± 540^†§^	3423 ± 573^†§^	3421 ± 612^†§^	3445 ± 605^†§^

BMI, body mass index.

Data are mean +/− standard deviation or n (%).

*In a past pregnancy: past gravidic hypertension, past preeclampsia, past eclampsia.

°Systemic lupus erythematosus, cardiac disease, inherited thrombophilia, previous thromboembolism event, chronic kidney disease, Crohn disease.

^†^Tests BMI classes versus BMI reference (normal weight); p < 0.05.

^§^Tests BMI class in Canada versus the same BMI class in France (France is the reference); p < 0.05.

**Figure 1:**
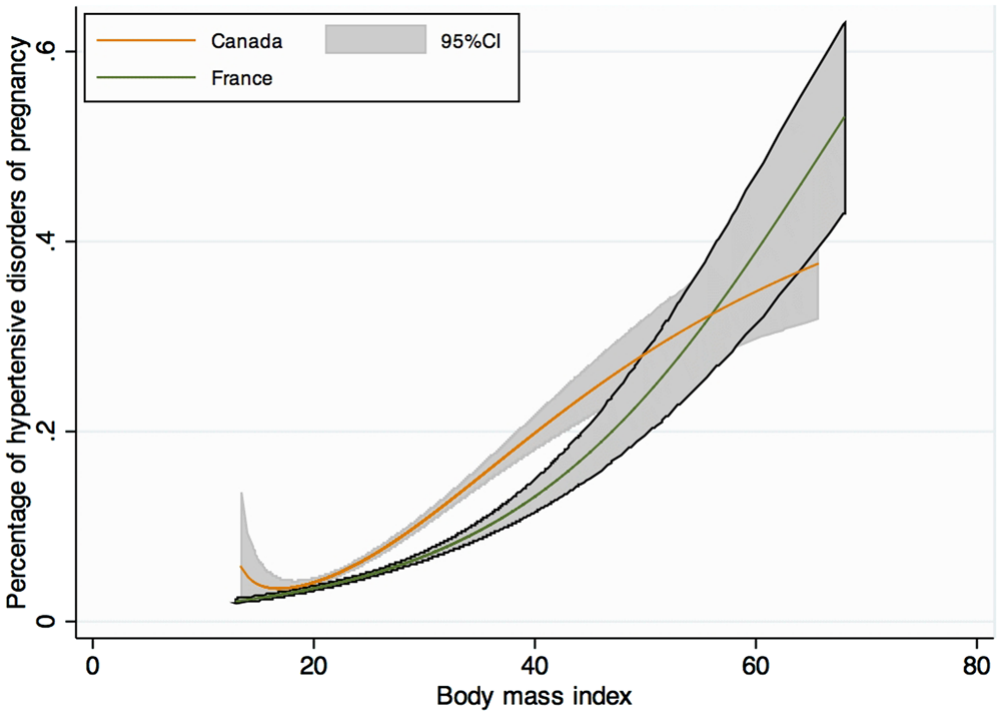
Crude risk of hypertensive disorders of pregnancy according to body mass index, modeled with fractional polynomials, and stratified by cohort.

**Figure 2:**
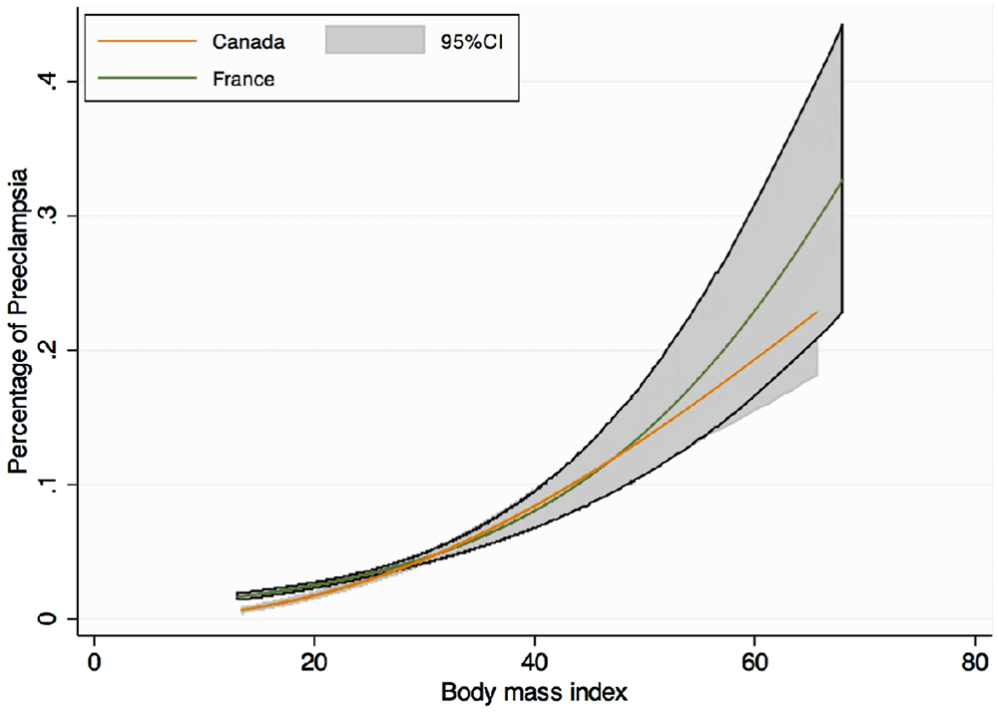
Crude risk of preeclampsia according to body mass index, modeled with fractional polynomials, and stratified by cohort.

**Figure 3:**
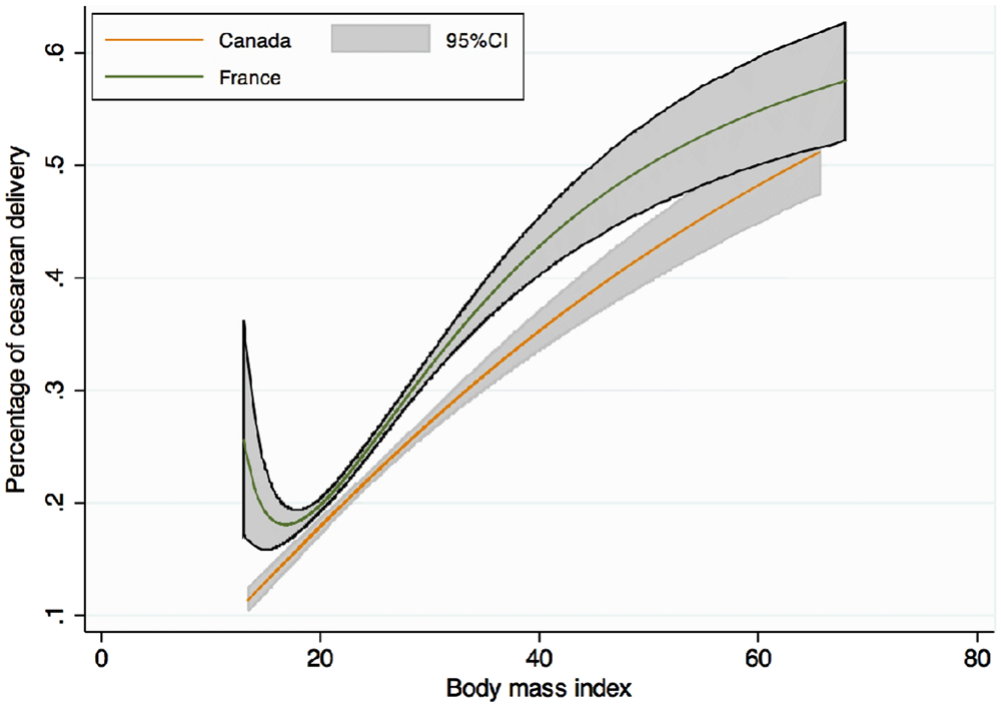
Crude risk of cesarean delivery according to body mass index, modeled with fractional polynomials, and stratified by cohort.

**Figure 4:**
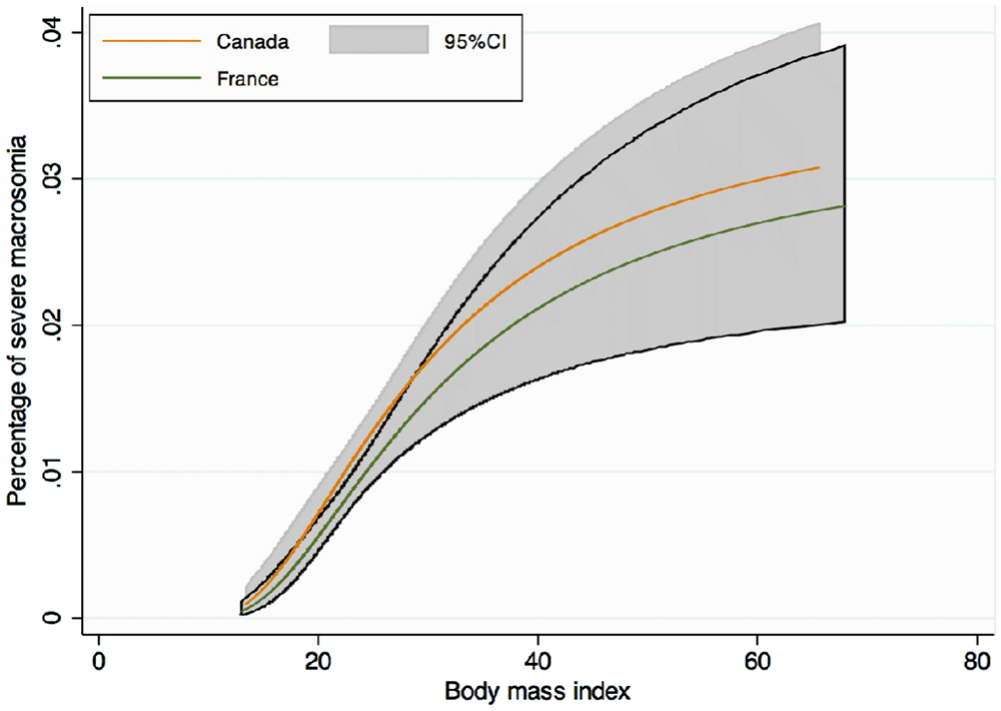
Crude risk of severe macrosomia (birth weight > 4500 g) according to body mass index, modeled with fractional polynomials, and stratified by cohort.

Maternal complications of pregnancy are presented in Tables 2 and 3 and a graph of the frequency of each complication according to BMI level stratified by country is presented in Figs 1–4. As the occurrence of venous thromboembolism and stillbirth was low, we did not compute a graph of those two outcomes that would not be very informative, due to very wide confidence intervals

**Table 2:** Maternal complications of pregnancy.

	FRENCH COHORT						CANADIAN COHORT					
	Underweight	Normal Weight	Overweight	Obesity			Underweight	Normal Weight	Overweight	Obesity		
				Class I	Class II	Class III				Class I	Class II	Class III
Hypertensive disorders of pregnancy	1303 (4.6)						1629 (7.4)^§^					
n (%)	64 (2.9)^†^	657 (3.8)	279 (5.6)^†^	138 (8.3)^†^	67 (12.3)^†^	37 (14.7)^†^	32 (5.9)^§^	618 (4.8)^§^	434 (8.4)^†§^	281 (13.0)^†§^	156 (18.1)^†§^	108 (21.4)^†§^
Crude RR	0.9 [0.7–1.1]	1	1.6 [1.5–1.7]	2.4 [2.2–2.6]	3.3 [2.9–3.8]	3.9 [3.4–4.5]	p interaction = 0.09 and 0.07					
Adjusted RR**	0.9 [0.7–1.2]	1	1.4 [1.3–1.6]	2.1 [1.9–2.4]	2.6 [2.3–3.1]	2.8 [2.3–3.3]						
Adjusted RR obtained with fractional polynomials*	0.7 [0.7–0.7]	1	1.5 [1.5–1.6]	2.2 [2.0–2.5]	3.0 [2.7–3.4]	3.5 [3.3–4.0]						
Preeclampsia^α^	911 (3.2)						697 (3.2)					
n (%)	49 (2.3)	474 (2.7)	182 (3.6)^†^	85 (5.1)^†^	44 (8.1)^†^	24 (9.5)^†^	13 (2.4)	274 (2.1)^§^	174 (3.4)^†^	122 (5.7)^†^	61 (7.1)^†^	53 (10.5)^†^
Crude RR	0.9 [0.7–1.1]	1	1.4 [1.3–1.6]	2.2 [1.9–2.5]	3.0 [2.5–3.6]	4.1 [3.3–5.1]						
Adjusted RR**	0.9 [0.6–1.2]	1	1.3 [1.2–1.5]	2.1 [1.7–2.4]	2.3 [1.8–2.9]	2.8 [2.1–3.6]	p interaction = 0.26 and 0.06					
Adjusted RR obtained with fractional polynomials*	0.7 [0.7–0.7]	1	1.4 [1.4–1.5]	1.9 [1.7–2.1]	2.5 [2.1–2.8]	3.2 [2.6–3.9]						
Venous thromboembolism	51 (0.2)						50 (0.2)					
n (%)	7 (0.3)	26 (0.2)	12 (0.2)	3 (0.2)	2 (0.4)	0 (0)	2 (0.4)	29 (0.2)	7 (0.1)	8 (0.4)	2 (0.2)	2 (0.4)
Crude RR	1.9 [0.9–3.9]	1	1.0 [0.6–1.7]	1.5 [0.8–2.9]	1.5 [0.5–4.1]	1.4 [0.3–5.7]						
Adjusted RR***	1.1 [0.5–2.8]	1	1.1 [0.6–1.8]	1.5 [0.8–2.8]	0.9 [0.3–2.6]	0.9 [0.2–3.8]	p interaction = 0.09 and 0.15					
Adjusted RR obtained with fractional polynomials*	1.0 [0.8–1.1]	1	1.1 [0.9–1.3]	1.1 [0.8–1.6]	1.2 [0.7–2.1]	1.3 [0.6–2.8]						

Data are n (%); RR: risk ratios.

When interaction exists between cohort and studied outcome, stratified analysis is reported with different RR in each cohort. In the absence of interaction, a single RR is reported and presented in the France group as an RR.

^†^Tests BMI classes versus BMI reference (normal weight) [only reported for n (%)]; p < 0.05.

^§^Tests BMI class in Canada versus the same BMI class in France (France is the reference); p < 0.05.

^α^Includes preeclampsia^,^ severe preeclampsia and eclampsia.

*Type 1 and Type 2 diabetes were excluded. Adjustment was made for ethnicity, maternal age and prior gestational diabetes.

**Adjusted for ethnicity, maternal age, smoking status, parity, history of hypertensive disorders of pregnancy, chronic hypertension, systemic lupus erythematosus, cardiac disease, inherited thrombophilia, chronic kidney disease, Crohn disease, type 1 diabetes, type 2 diabetes.

***Adjusted for ethnicity, maternal age, inherited thrombophilia and previous thromboembolism event.

**Table 3:** Other maternal complications of pregnancy.

	FRENCH COHORT						CANADIAN COHORT					
	Underweight	Normal Weight	Overweight	Obesity			Underweight	Normal Weight	Overweight	Obesity		
				Class I	Class II	Class III				Class I	Class II	Class III
Stillbirth	165 (0.6)						63 (0.3)^§^					
n (%)	7 (0.3)	78 (0.5)	33 (0.7)	14 (0.8)^†^	7 (0.9)		1 (0.2)	33 (0.3)^§^	18 (0.4)^§^	4 (0.2)^§^	7 (0.5)	
Crude RR	0.7 [0.3–1.4]	1	1.4 [1.0–2.0]	1.4 [0.9–2.3]	2.0 [1.2–3.5]		p interaction = 0.63 and 0.45					
Adjusted RR*	0.7 [0.3–1.7]	1	1.5 [1.0–2.1]	1.5 [0.8–2.6]	2.3 [1.2–4.2]							
Adjusted RR obtained with fractional polynomials*	0.8 [0.7–0.9]	1	1.3 [1.1–1.4]	1.6 [1.2–2.0]	2.2 [1.4–3.6]							
Cesarean delivery	6737 (23.8)						4975 (22.6)^§^					
n (%)	411 (18.9)^†^	3707 (21.4)	1378 (27.5)^†^	571 (34.4)^†^	217 (39.8)^†^	112 (44.4)^†^	104 (19.0)	2458 (19.2)^§^	1319 (25.5)^†§^	621 (28.8)^†§^	279 (32.3)^†§^	194 (38.4)^†^
Crude RR	0.9 [0.8–0.9]	1	1.3 [1.2–1.3]	1.5 [1.5–1.6]	1.7 [1.6–1.8]	1.9 [1.8–2.1]	p interaction = 0.15 and 0.09					
Adjusted RR**	0.9 [0.9–0.9]	1	1.2 [1.1–1.2]	1.2 [1.2–1.3]	1.3 [1.2–1.4]	1.5 [1.3–1.6]						
Adjusted RR obtained with fractional polynomials*	0.8 [0.8–0.8]	1	1.3 [1.2–1.3]	1.5 [1.4–1.5]	1.7 [1.6–1.8]	1.9 [1.8–2.1]						
Birth weight > 4000 g	2011 (7.1)						2094 (9.5)^§^					
n (%)	93 (4.3)^†^	1,139 (6.6)	457 (9.2)^†^	166 (10.0)^†^	49 (9.0)^†^	23 (9.1)	25 (4.6)^†^	991 (7.8)^§^	586 (11.3)^†§^	288 (13.4)^†§^	127 (14.7)^†§^	77 (15.3)^†§^
Crude RR	0.6 [0.5–0.8]	1	1.4 [1.3–1.5]	1.6 [1.5–1.8]	1.7 [1.5–1.9]	1.8 [1.5–2.1]	p interaction = 0.15 and 0.20					
Adjusted RR***	0.7 [0.5–0.8]	1	1.4 [1.3–1.5]	1.6 [1.4–1.7]	1.6 [1.4–1.9]	1.7 [1.4–2.0]						
Adjusted RR obtained with fractional polynomials*	0.6 [0.6–0.6]	1	1.4 [1.3–1.4]	1.7 [1.6–1.8]	1.8 [1.7–2.0]	2.0 [1.9–2.2]						
Birth weight > 4500 g	240 (0.9)						272 (1.2)^§^					
n (%)	10 (0.5)	121 (0.7)	54 (1.1)^†^	31 (1.9)^†^	13 (2.4)^†^	4 (1.6)	3 (0.6)	117 (0.9)^§^	77 (1.5)^†^	44 (2.0)^†^	21 (2.4)^†^	10 (2.0)^†^
Crude RR	0.6 [0.4–1.1]	1	1.6 [1.3–2.0]	2.4 [1.9–3.2]	2.9 [2.0–4.1]	2.2 [1.3–3.7]	P interaction = 0.96 and 0.90					
Adjusted RR***	0.7 [0.4–1.1]	1	1.5 [1.2–1.9]	2.2 [1.7–2.8]	2.7 [1.9–3.8]	2.0 [1.1–3.5]						
Adjusted RR obtained with fractional polynomials*	0.5 [0.4–0.6]	1	1.6 [1.4–1.8]	2.0 [1.7–2.4]	2.4 [1.9–2.9]	2.7 [2.1–3.4]						

Data are n (%); RR: Risk ratios. When interaction exists between cohort and studied outcome, stratified analysis is reported with different RR in each cohort. In the absence of interaction, a single RR is reported and presented in the France group as an RR.

^†^Tests BMI classes versus BMI reference (normal weight) [only reported for n (%)]; p < 0.05.^§^Tests BMI class in Canada versus the same BMI class in France (France is the reference); p < 0.05.

*Adjusted for ethnicity, maternal age, gestational diabetes, type 1 and type 2 diabetes, history of hypertensive disorders of pregnancy and inherited thrombophilia.

**Adjusted for ethnicity, maternal age, occurrence of hypertensive disorders during the pregnancy, suspicion of intrauterine growth restricted, suspicion of fetal macrosomia, prior cesarean delivery, and breech presentation.

***Adjusted for ethnicity, maternal age, parity, gestational diabetes, type 1 and type 2 diabetes.

Hypertensive disorders of pregnancy (HDP) were significantly more frequent amongst Canadian women (7.4% vs. 4.6%, p < 0.001). A graph of the PF modeling, which computes the frequency of HDP in both cohorts according to BMI level, is presented in Fig. 1. Increasing BMI categories was associated with an increased HDP rate irrespective of the cohort. Testing for cohort interaction was not significant, neither in the crude nor in both adjusted analyses (Categorical BMI modeling of PF modeling) (p = 0.09 and p = 0.07). Therefore, aRR did not differ between cohorts for HDP according to BMI categories. Nevertheless, after adjustment for confounders (ethnicity, maternal age, smoking status, parity, history of hypertensive disorders of pregnancy, chronic hypertension, systemic lupus erythematosus, cardiac disease, inherited thrombophilia, chronic kidney disease, Crohn disease, type 1 diabetes, type 2 diabetes), the values of aRR in both cohorts significantly increased with increasing BMI category to a maximum of 3.5 [95% CI; 3.3–4.0] for class III obesity.

The frequency of preeclampsia, severe preeclampsia and eclampsia according to BMI categories was similar in both cohorts (Fig. 2). As for HDP, testing for cohort interaction was not significant (p = 0.26 and p = 0.06). Nevertheless, obese women were significantly at higher risk of preeclampsia (mild, severe and eclampsia) than normal weight women with increasing aRR by BMI category in both cohorts.

Regarding venous thromboembolism, similar rates were observed in France and Canada (0.2%). The incidence of venous thromboembolic disorders remained the same and was independent of the cohort and the BMI category. No significant interaction between BMI and country cohort was observed (p = 0.9) and aRR were not significantly different between an obese and non-obese cohort after appropriate adjustments on ethnicity, maternal age, inherited thrombophilia and previous thromboembolism event were considered.

Stillbirth was more frequent in France than Canada (0.6% vs 0.3%; p < 0.05) and a trend toward an increase frequency of stillbirth among obese patient was observed. The relationship between stillbirth and BMI was not different across both cohorts (p = 0.63 and p = 0.45). After adjustment on ethnicity, maternal age, type 1 and type 2 diabetes, history of hypertensive disorders of pregnancy and inherited thrombophilia, increasing BMI category was associated with an increased risk of stillbirth.

Rates of caesarean section were lower in Canada than in France (22.6% vs. 23.8%, p < 0.001) (Fig. 3). In each BMI category Canadian rates of caesarean almost corresponded to the corresponding lower BMI category in the French group. However, no significant interaction between cohort and BMI was observed in either crude or adjusted analyses (adjustment on ethnicity, maternal age, occurrence of hypertensive disorders during the pregnancy, suspicion of intrauterine growth restricted, suspicion of fetal macrosomia, prior cesarean delivery, and breech presentation) (p = 0.15 and p = 0.1), and a single aRR was therefore computed.

Macrosomia (birth weight > 4000 g) was 1.5 times more frequent in Canada than in France (9.5% vs. 7.1%, p < 0.001), overall and across BMI categories. For macrosomia or severe macrosomia (birth weight > 4500 g) no significant interaction between BMI and country cohort was observed (p = 0.15 and p = 0.95) (Fig. 4). Adjusted RR were similar among cohorts, but remained strongly associated with increasing BMI, up to a risk of 2.7 [95% CI; 2.1–3.4] for Class III obesity, after adjustment on covariates (ethnicity, maternal age, parity, type 1 and type 2 diabetes).

## Discussion

Following our analysis, we find that obesity was associated, both in French and Canadian cohort, with an increased risk of hypertensive disorders of pregnancy, preeclampsia, stillbirth, cesarean delivery and macrosomia compared to patients with a normal BMI. Even if the prevalence of these complications of pregnancy was higher in Canada than in France, the relationship between BMI and those outcomes was similar in both cohorts.

In both cohorts, the prevalence of obesity was similar to the one expected both in France according to a French perinatal study in 2010 (9.9%)^7^, and in Canada, according to Canadian population data (16%)^6^. However, we could not stand that each cohort is representative of the country they are issued, as they represent high-risk pregnancies/deliveries coming from tertiary care centers. Baseline characteristics in Table 1 demonstrated that French women appeared to be at slightly higher risk of pregnancy complications than Canadian women. This might be explained by differences in the health care system of both countries. The Canadian cohort only included women from the province of Quebec, where all citizens are covered by health care insurance. In France, citizens also benefit from a public national health system. However, access to medical care is greatly reduced in Quebec. Only 74.2% of the population in Quebec reported having access to a general practitioner, relative to an almost 100% in France^17^. Furthermore, delays to obtain an appointment in Quebec may also be very long. This implies that many obese women may not have a regular medical follow-up, and pregnancy may become the period where chronic diseases are first diagnosed. Therefore, obese women may be unknowingly afflicted with chronic hypertension or chronic diabetes, and wrongly classified during pregnancy as having gestational hypertension or gestational diabetes. This may artificially increase the rate of pregnancy complications amongst Canadian women. Such hypothesis is supported by results in Table 1, where rates of chronic hypertension, and type 1 diabetes are slightly lower in Canada than in France.

We confirmed that obesity is a strong risk factor for the development of various complications of pregnancy in both cohorts. The effect sizes were consistent with previous publications^18–21^, and the trend in increasing risk ratios with increasing obesity classes remained present in both cohorts similarly for each studied outcome, except for venous thromboembolism.

Hypertensive disorders of pregnancy (HDP) are relevant markers of adverse maternal and fetal health. The rate of hypertensive complications in pregnancy was higher in Canada, irrespective of BMI categories, whereas the rate of chronic hypertension was lower in Canada. Again, insufficient follow-up of non-pregnant women by lack of general practitioners, may contribute to artificially increase the rate of wrongly diagnosed “gestational hypertension” instead of actual chronic hypertension. This hypothesis is also supported by similar rate of preeclampsia (mild, severe and eclampsia) across Canada and France. The difference might also be partly explained by different guidelines and diagnostic criteria between the two countries^22, 23^. The increased rate of gestational hypertension might also be related to difference in food habits, Canadians having food habits closer to those of American than European lifestyles. Even if the rate of hypertensive disorders in pregnancy were different in Canada and France, the RR and aRR could be represented as a unique function of risk due to similar trend of risk increasing with BMI.

We were surprised to find that the prevalence of venous thromboembolic disorders appeared similar across BMI categories and also across cohorts. However, the number of events were low in both cohorts and an underreporting bias is still possible; especially in the French cohort as it is not issued from a RCT were patients are followed intensively. Even if crude RR tended to increase slightly with increasing BMI, no relationship was found between BMI and venous thromboembolism after adjusting for confounders in the multiple logistic regression analyses. This might also reflect the appropriate adjustment for confounding factors, such as inherited thrombophilias or prior thromboembolic events.

The relationship between stillbirth and BMI categories persisted after adjusting at a similar level between France and Canada, confirming the independent influence of obesity in this situation. However, aRR were slightly lower than previously reported^21^, either because of appropriate adjustment in our study, or because possible misclassification of terminations of pregnancy. Unlike previous study we took great care to adjust on known confounder of literature. An explanation of the increase incidence of stillbirth in France might rely in the fact that French women where at baseline at slightly higher risk of pregnancy complications.

Regarding caesarean section, we confirmed the known association with obesity^24^. Lower rates in Canada could be related to a greater experience in managing obese patients leading to reduce rates of elective cesarean delivery for the single indication of being obese. However, this would have involved extended analyses and would have required knowing the indication for surgical delivery, which was not available in the databases. In both groups, class III obesity carried a two-fold risk of cesarean delivery compared to normal BMI women.

Regarding macrosomia and severe macrosomia, no measurement bias could explain the difference in rates between countries. Macrosomia was 1.5 to 2 times more frequent in Canada irrespective of BMI categories, but the relationship between macrosomia and BMI was the same in both cohorts, showing that the mechanism of increase are the same. Higher frequency of macrosomia in Canada is probably related to food habits, but it might also be partly explained by genetic factors.

Global results of our work show that the relationship between obesity and various complication of pregnancy are the same across France and Canada even if obesity is more prevalent in Canada and some complication more frequently observed. These results indicate that we can predict an increase in the complication of pregnancy rate in France if obesity continues to increase in France as it does in Canada. Therefore, the current situation in Canada would correspond to the situation of France in about 15 years if no prevention were to be undertaken. These results could help inform professionals and decision makers about obstetrical health risk and neonatal complications caused by the emergence of obesity in pregnancy. This would imply promoting primary prevention of obesity in women before pregnancy or secondary prevention via a reduction in complications in obese pregnant women.

Our study has the following strengths: it included a large number of patients in the same period of time, and diagnoses were prospectively made in both cohorts with the same criteria. Canadian data included a large representative sample of hospitals in the province of Quebec and was not influenced by the QUARISMA intervention as they came from the observational arm of the trial. On the other hand, limitations of this study might be related to several parameters. Some particular outcomes were not accessible making it difficult to undertake a good and reliable comparison. For example, gestational diabetes, initially studied, was finally waived out according to doubts of misclassification biases. French and Canadian have different guidelines, used different methods of screening with different thresholds and at a different time. As data came from a database extraction, the risk of misdiagnosis or inaccurate data still exists, but this is lowered by the fact that each diagnosis is coded by physicians during patients’ follow-up. Another limitation of our study is that some difference between obese may not have properly been adjusted on. As we worked on database, some information such as medical treatment for obese women, diet, and lifestyle or socio-demographic condition were not available and could not have been taken as adjustment parameter. Finally, as Canadian data came from a planned RCT, other comparisons than those planned in the original study, could not be undertaken.

In conclusion, obesity was associated with adverse maternal outcome in both France and Canada. Risks associated with obesity gradually increased with classes of obesity. The strength of the association was similar between the two cohorts for every studied outcome even though the prevalence of obesity and of some complication was higher in Canada.
